# Mechanistic Features of Nanodiamonds in the Lapping of Magnetic Heads

**DOI:** 10.1155/2014/326427

**Published:** 2014-06-22

**Authors:** Xionghua Jiang, Zhenxing Chen, Joy Wolfram, Zhizhou Yang

**Affiliations:** ^1^School of Chemistry and Chemical Engineering, Sun Yat-sen University, Guangzhou 510275, China; ^2^National Center for Nanoscience & Technology of China, Beijing 100190, China; ^3^Department of Emergency, Jinling Hospital Medical School of Nanjing University, Nanjing 210000, China

## Abstract

Nanodiamonds, which are the main components of slurry in the precision lapping process of magnetic heads, play an important role in surface quality. This paper studies the mechanistic features of nanodiamond embedment into a Sn plate in the lapping process. This is the first study to develop mathematical models for nanodiamond embedment. Such models can predict the optimum parameters for particle embedment. From the modeling calculations, the embedded pressure satisfies *p*_0_ = (3/2)·(*W*/*πa* ^2^) and the indentation depth satisfies $\delta =k_{1}\sqrt{P/HV}$. Calculation results reveal that the largest embedded pressure is 731.48 GPa and the critical indentation depth *δ* is 7 nm. Atomic force microscopy (AFM), scanning electron microscopy (SEM), and Auger electron spectroscopy (AES) were used to carry out surface quality detection and analysis of the disk head. Both the formation of black spots on the surface and the removal rate have an important correlation with the size of nanodiamonds. The results demonstrate that an improved removal rate (21 nm*·*min^−1^) can be obtained with 100 nm diamonds embedded in the plate.

## 1. Introduction

Nanodiamonds refer to diamonds with a diameter equal to or less than 100 nm. These particles not only exhibit properties of diamonds, but also have special nanoparticle characteristics, such as small size, unique surface properties, and macroscopic quantum tunneling [1, 2]. In recent years, many scholars have performed research on the stability, shape, and particle size distribution of nanodiamonds [3, 4]. Nanodiamonds aggregate easily, so studies on dispersion of nanodiamonds are especially important. In particular, recent studies have focused on the thermodynamic, dynamic, and aggregate stability of nanodiamonds [5–8]. Many scholars have performed valuable research on the dispersion of nanodiamonds in aqueous systems [9–12]. However, because of the metallic properties of magnetic head materials, the lapping process requires nonaqueous systems to avoid potential chemical corrosion. Dispersion of nanodiamonds in nonaqueous systems directly affects the stability of the particles embedded in the plate, thus affecting the surface lapping quality of the magnetic head. Solid content, oil density, and surface modifying agents of nanodiamonds are considered to be the main factors affecting the dispersion in nonaqueous systems [13, 14]. Due to hard aggregation composed of Van Edward forces and hydrogen bonds and soft aggregation caused by the surface atomic binding force, the methods to improve nanodiamond stability include the following: (i) adding a surfactant that effects surface absorption or chemical reactions of nanodiamonds, (ii) using polymer nanodiamond powder instead of a monomer to avoid particles getting in close proximity to each other, and (iii) ultrasonic dispersion [15–17]. When nanodiamonds with good dispersion are used, the slurry is harder, has higher heat transfer and endurance, and improves chemical stability [18–21]. When nanodiamonds are used in the lapping of magnetic heads, the surface roughness can be greatly reduced [22–25]. Some scholars believe that the mechanism of improvement is due to the existing ball bearing effect between the spherical nanodiamond and the Sn plate, where rolling and gliding friction coexist. The nanodiamonds can significantly increase lapping endurance and reduce the friction effect [26, 27]. Other researchers believe that the improved performance is due to the existing boundary lubrication among the nanodiamonds, the magnetic heads, and the oil, all of which greatly reduce friction [28]. There are few studies on the process of nanodiamond embedment into a Sn plate in the lapping process. In this paper, the mechanistic features of nanodiamond embedment are analyzed and for the first time mathematical formulas are established to model this process. These models encompass two key factors: the pressure of nanodiamonds embedded into the plate and the embedment depth. These parameters impact the lapping surface of the magnetic head, especially the formation of black spots and the material removal rate. Therefore, the models can be used to calculate optimum depth and pressure values for the embedment process.

**Figure 1:**
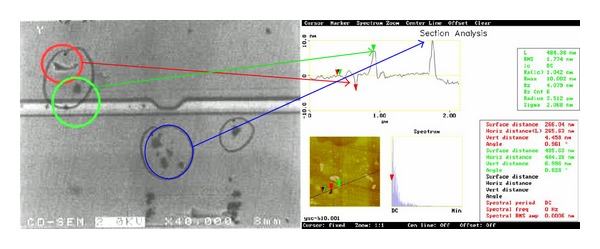
(a) Scanning electron microscopy (SEM) images of black spots, obtained with Hitachi S4800 high resolution SEM with Vacc 2.0 kV, ×40 K. (b) Atomic force microscopy (AFM) images of the corresponding black spots from the SEM images, obtained with PSIA AFM from PARK Company, Korea, using tapping mode and a scan size of 10 × 40 *μ*m.

**Figure 2:**
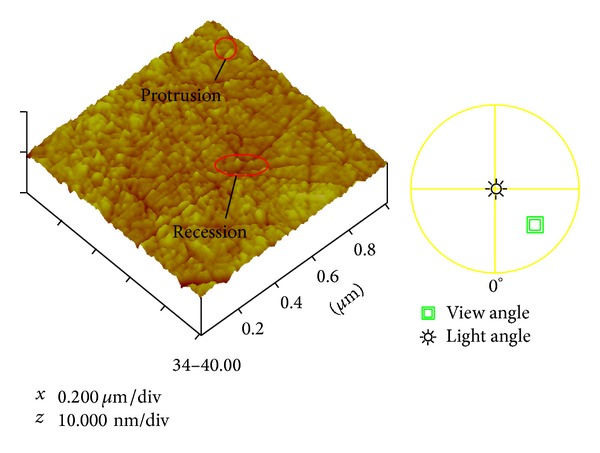
3D AFM images detected of the protrusion (6.966 nm) and recession (4.458 nm) of black spots, obtained with PSIA AFM from PARK Company, Korea, using tapping mode and a scan size of 2 *μ*m.

As can be seen in Figures 1 and 2, scanning electron microscopy (SEM) and atomic force microscopy (AFM) images reveal black spots on the surface of magnetic heads. Black spots refer to round areas with white reflection that are usually bigger than 10 nm in diameter and have diamond-like protrusions. Since the capacity of a Sn plate to hold nanodiamonds is limited, the pressure and embedment depth of nanodiamonds are very important. When the nanodiamonds are exposed to less pressure, the embedment depth is shallow, and the number of embedded particles is less. In this case, it is hard for the magnetic head materials to touch the diamonds in the lapping process. The nanodiamonds embedded into the plate surface can also easily fall off and flow along with the slurry. In both cases, this will cause a low removal rate. When the nanodiamonds are under more pressure, the embedded depth will be deeper and the amount of embedded particles will increase, resulting in deformation of the Sn plate and defects in the head surface, including scratches, protrusions, recession, and black holes. Auger electron spectroscopy (AES) E1127-08 was used to do further research on the composition of the black spots, as shown in Table 1. The experimental data confirmed that the carbon content of the region with black spots is much higher than that of a normal region.

## 2. Establishment of a Model for Nanodiamond Embedment

### 2.1. Pressure Analysis

**Figure 3:**
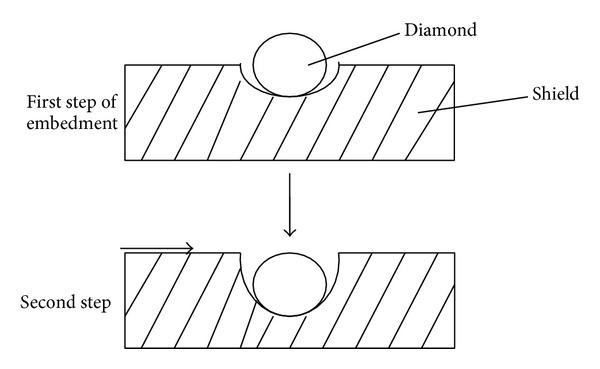
Modeling of the embedment of a single nanodiamond.

In the first step of the model, the effect of a single diamond was considered. A spherical diamond with a diameter of 100 nm was employed without movement or lubrication. The embedment was accomplished in two steps, as shown in Figure 3. The process evolved from elastic deformation to elastic-plastic deformation to plastic deformation and finally reached maximum plastic deformation. When the tough circle radius is the same as the diamond radius, the diamond is embedded into the shield.

Three values should be obtained for the model: (i) maximum elastic deformation (ii), maximum plastic deformation, and (iii) corresponding pressure. In the first step, plastic deformation takes place and maximum deformation appears when half of the diamond is embedded into the shield. Based on the classical plastic contact equation and considering adherent energy [29], we obtain

(1) $$\begin{array}{c} P+2\pi wr=\pi a^{2}H, \end{array}$$

where *P* refers to load, *w* to adherent energy, *r* to diamond radius, *a* to tough circle radius, and *H* to hardness. *ω* can be obtained from

(2) $$\begin{array}{c} w=2\varphi {\left(\gamma_{a}\gamma_{b}\right)}^{1/2}, \end{array}$$

where *γ*_*a*_ and *γ*_*b*_ refer to the surface force of shield and diamond, respectively, and *φ* refers to the related constant. When we regard *r* as the radius of circle in the case of maximum deformation [30], we get

(3) $$\begin{array}{c} P_{1}=\pi r^{2}H-4\pi \varphi r{\left(\gamma_{a}\gamma_{b}\right)}^{1/2}, \end{array}$$

where *P*_1_ refers to the pressure when plastic deformation begins.

In the second step, whole embedment takes place, based on classical contact mechanics theory [31]:

(4) $$\begin{array}{c} a={\left(\frac{3WR_{0}}{E^{\prime }}\right)}^{1/2}-{\left(\frac{3}{2}\cdot \frac{WR}{E^{\prime }}\right)}^{1/2},\\ \frac{1}{R}=\frac{1}{R_{1}}+\frac{1}{R_{2}},\\ \frac{1}{E^{\prime }}=\frac{1}{2}\left(\frac{1-\nu_{1}^{2}}{E_{1}}+\frac{1-\nu_{2}^{2}}{E_{2}}\right), \end{array}$$

where *E*_1_ = 143 GPa, *E*_2_ = 1141 GPa, *ν*_1_ = 0.23, and *ν*_2_ = 0.07; thus *E*′ = 226.84 GPa. For a spherical diamond, *R*_2_ = *R*_0_ and *R*_1_ = *∞* for plane; thus *R* = *R*_0_, and the largest pressure [32] is

(5) $$\begin{array}{c} p_{0}=\frac{3}{2}\cdot \frac{W}{\pi a^{2}}. \end{array}$$

### 2.2. Indentation Depth Analysis

**Figure 4:**
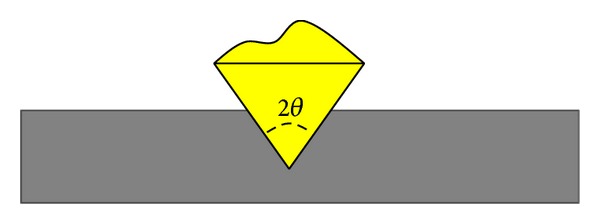
Modeling of the indentation depth of a single nanodiamond.

As shown in Figure 4, according to the test method of microhardness, the indentation depth *δ* is determined by hardness and load as follows [33]:

(6) $$\begin{array}{c} \delta^{2}\propto \frac{P}{HV}. \end{array}$$

From the above formula, we have $\delta =k_{1}\sqrt{P/HV}$, where *k*_1_ is a constant related to the shape of the diamond.

### 2.3. Removal Rate Analysis

#### 2.3.1. Plastic Deformation [34]

(7) $$\begin{array}{c} V=K_{1}\cdot P\cdot H^{-1}\cdot L, \end{array}$$

where *V* is removal volume, *P* is pressure, *H* is hardness of removed materials, *L* is sliding distance, and *K*_1_ is a universal parameter determined by the friction coefficient, the diamond shape, plate charging quality, toughness, and plastic models of the magnetic heads.

#### 2.3.2. Crack Deformation [34]

(8) $$\begin{array}{c} V=K_{2}\cdot P^{5/4}\cdot H^{-1/2}\cdot T^{-3/4}\cdot L, \end{array}$$

where *P* is pressure, *H* is hardness, *T* is crack toughness, *L* is sliding distance, and *K*_2_ is a universal parameter determined by the shape and radius of the diamond.

## 3. Experiments

### 3.1. Material Preparation

**Figure 5:**
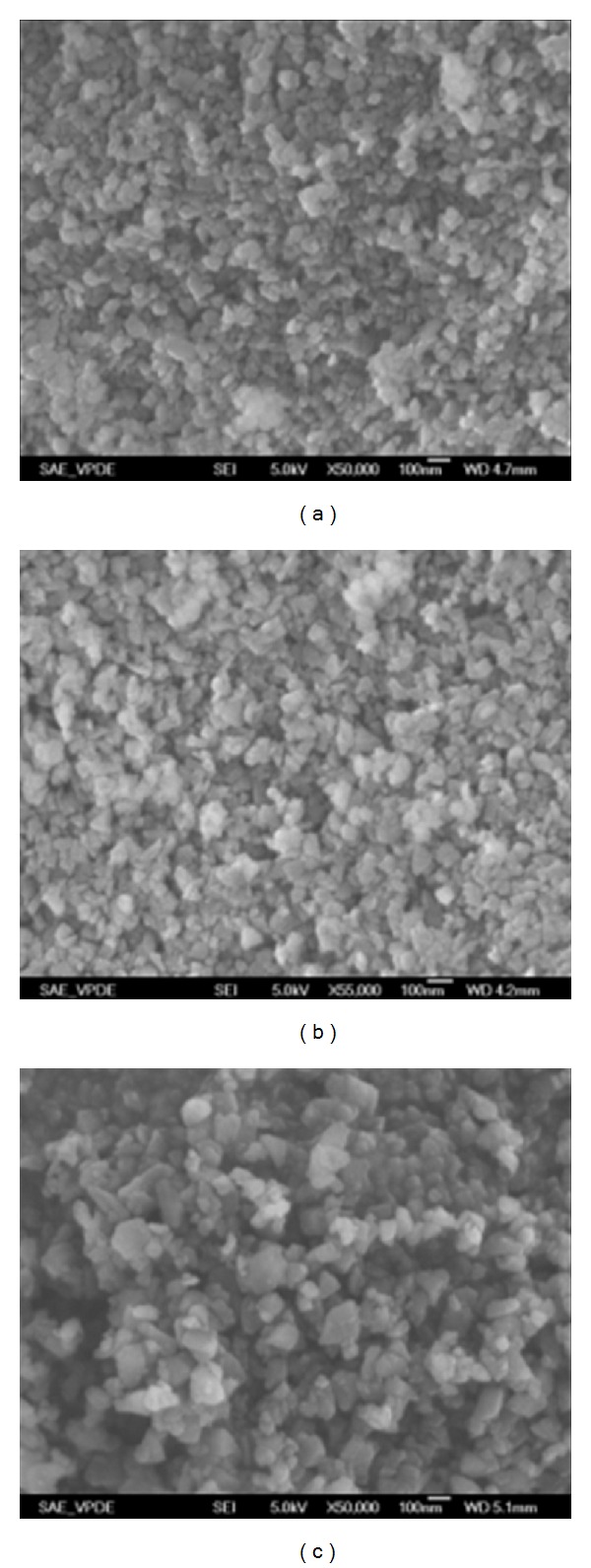
SEM pictures of diamonds. (a) 50 nm diamonds from 1/20, CKK Co., USA (×50 K). (b) 70 nm diamonds from 1/15, CKK Co., USA. (×50 K). (c) 100 nm diamond from 1/10, CKK Co., USA. (×50 K).

Three types of nanodiamonds extracted from CKK slurry with different radii, which were used for head lapping plate preparation, were imaged with a Hitachi S4800 high resolution SEM, with Vacc 5.0 kV (Figure 5).

### 3.2. Machine and Embedding Process

**Figure 6:**
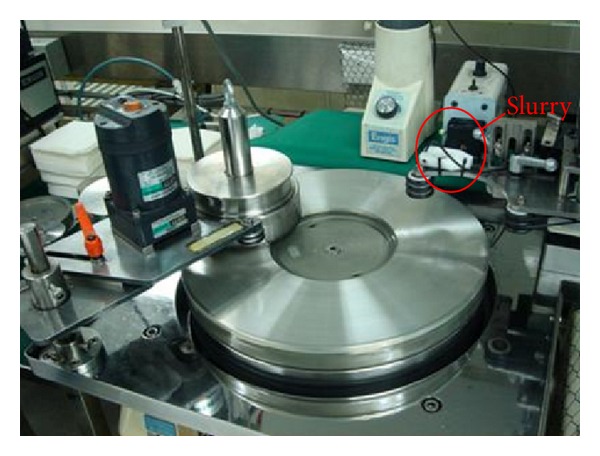
Lapping machine.

A HYPREZ lapping machine from Engis Company, Japan, was used. As shown in Figure 6, the Sn plate and the fixture were rotated with different motors through transmission belts to keep the lapping speed adjustable. Freeze water was used to ensure constant temperature by removing heat. The plate and the ring were rotated at a fixed speed in the same direction. A magnetic bar (PMR, produced by TDK Co.) was fixed on the ring with rubber. Each magnetic bar had 81 heads (sliders) and a size of 69.6 × 1.235 × 0.23 mm. Slurry was added from a capillary tube and subjected to continuous stirring.

The base material of the plate was Sn. The rotation speed was set at 30 rpm*·*min^1^. The ring rotation speed was set at 25 rpm*·*min^−1^, the weight was 6 kg, and the shaving time was 25 min. The lubrication was AM-ZX-60 from Engis Co. Removal rates were measured by calculating the resistance of magnetic heads before and after lapping by using a SSCL-F precision magnetic test machine made by TDK, Japan.

### 3.3. Results and Discussion

#### 3.3.1. Pressure and Indentation Depth

The magnetic head is composed of an AlTiC hard and brittle ceramic substrate with several micrometer thick metal read and write pole tips (1.235 × 0.7 × 0.23 mm). The substrate is composed of 64% Al_2_O_3_ and 36% TiC (volume ratio), achieved by sintering. The mechanical properties are shown in Table 2. The composite ceramic of AlTiC has a large density, with a hardness of 23.7 GPa, which is higher than that of the pure ceramic Al_2_O_3_ (about 19 GPa). Therefore, the addition of TiC can improve the mechanical performance of the magnetic head substrate.

From (5), it can be calculated that the largest pressure put on the nanodiamonds is 731.48 GPa and the critical indentation depth *δ* is 7 nm. Namely, when the indentation depth is less than 7 nm, plastic deformation occurs, while crack deformation takes place at depth of more than 7 nm.

**Figure 7:**
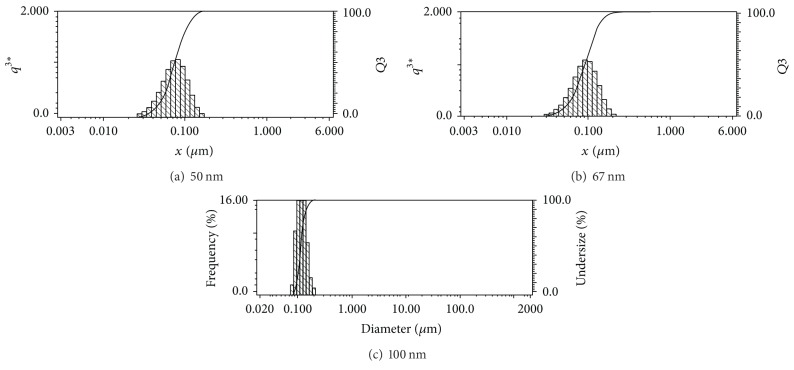
Particle size distribution of nanodiamonds. (a) 50 nm diamonds from 1/20, CKK Co., USA. (b) 70 nm diamonds from 1/15, CKK Co., USA. (c) 100 nm diamond from 1/10, CKK Co., USA.

In the lapping process, the average diameter of the nanodiamonds was approximately 100 nm. And the particle size distribution of nanodiamonds, as shown in Figure 7, was tested by HORIBA LB-550 granulometer. The measured data showed that the scope of these three types of diameter distribution was almost the same, from 50 nm to 240 nm, except the principal distribution difference. The diamonds were suspended in slurry, which was mixed as the magnetic bar and plate rotated. The magnetic bar was pressed onto the plate with a loading plate and a pressing block. Under the shear force from the relative motion between the grinding plate and the magnetic bar, some of the diamonds became embedded into the plate. When a diamond is pressed into the plate and reaches a certain depth, the plate exerts an elastic deformation force on the diamonds that is much larger than the magnetic shear stress from the magnetic bar. Consequently, the diamonds are fixed onto the grinding plate, forming a cutting edge. Microcutting of the magnetic surface occurs due to the relative motion between the lapping plate and the magnetic bar, thereby obtaining a smooth machined surface. However, nanodiamonds are typically embedded onto the plate using random pressure, and the sizes of the diamonds usually differ, causing various indentation depths. This in turn causes variations in the magnetic bar surface cutting height, which can lead to the formation of microscratches. Therefore, the establishment of mathematical models, such as (5) and (6), that can predict the indentation depth of diamonds is necessary to avoid microscratches [35].

#### 3.3.2. Removal Rates

Equations (7) and (8) demonstrate that the removal volume will increase if the pressure (*P*) and sliding distance (*L*) are increased. The removal rate increases as the weight increases. When the plate speed is raised, *L* in unit lapping time increases, leading to an increase in removal rate. When these parameters are fixed, removal rates are primarily determined by the diameter of the nanodiamonds. In this experiment, nanodiamonds with three different diameters were used (100 nm, 75 nm, and 50 nm). The original data of the removal rates of magnetic heads in the lapping process is about 20 nm*·*min^−1^. As can be seen in Table 3, higher removal rates are obtained as the diameter of the nanodiamonds increases, thereby showing that the experimental result correlates with the model predictions. The removal rate is 21 nm*·*min^−1^ when 100 nm nanodiamonds were used in lapping process, which was higher than the other two types of diamonds.

## 4. Summary

A mechanistic model of nanodiamonds embedded into a Sn plate has been established in this paper. The pressure satisfies *p*_0_ = (3/2)·(*W*/*πa* ^2^). And the indentation depth satisfies $\delta =k_{1}\sqrt{P/HV}$. The calculations reveal that the largest embedded pressure is 731.48 GPa, and the critical indentation depth *δ* is 7 nm. A removal rate equation has also been established. The removal rate equation under plastic deformation satisfies *V* = *K*_1_ · *P* · *H* ^−1^ · *L* and under crack deformation satisfies *V* = *K*_2_ · *P* ^5/4^ · *H* ^−1/2^ · *T* ^−3/4^ · *L*. In conclusion, the lapping process can be predicted and the surface quality of magnetic heads can be improved by using these formulas.

## Figures and Tables

**Table 1:** Auger electron spectroscopy (AES) data for black spots.

Composition	Black spots	Normal shield area
C	43.60%	18.90%
Ni	44.70%	64.10%
Fe	11.70%	16.90%

**Table 2:** Mechanistic properties of magnetic head materials.

Material	HV (GPa)	*E* (GPa)	*K* (MPa*·*m^1/2^)
64% Al_2_O_3_ 36% TiC	23.7	390	3.77

**Table 3:** Comparison of removal rates of nanodiamonds with different diameters.

Nanodiamonds	Diameter (nm)	Removal rate (nm*·*min^−1^)
1/20 CKK	50	1.2
1/15 CKK	75	6.966
1/10 CKK	100	21
